# Genomic Diversity and Zoonotic Potential of *Brucella neotomae*

**DOI:** 10.3201/eid3001.221783

**Published:** 2024-01

**Authors:** Gilles Vergnaud, Michel S. Zygmunt, Roland T. Ashford, Adrian M. Whatmore, Axel Cloeckaert

**Affiliations:** Université Paris-Saclay, Institute for Integrative Biology of the Cell, Gif-sur-Yvette, France (G. Vergnaud);; Institut National de l’Agriculture, de l’Alimentation et de l’Environnement, Unité Mixte de Recherche Infectiologie et Santé Publique, Université de Tours, Nouzilly, France (M.S. Zygmunt, A. Cloeckaert);; Animal and Plant Health Agency, Weybridge, UK (R.T. Ashford, A.M. Whatmore)

**Keywords:** Brucella neotomae, bacteria, zoonoses, food safety, whole-genome sequencing, WGS, single-nucleotide polymorphism, SNP, Costa Rica, Great Salt Lake, Utah, United States, France, United Kingdom

## Abstract

After reports in 2017 of *Brucella neotomae* infections among humans in Costa Rica, we sequenced 12 strains isolated from rodents during 1955–1964 from Utah, USA. We observed an exact strain match between the human isolates and 1 Utah isolate. Independent confirmation is required to clarify B. neotomae zoonotic potential.

The genus *Brucella* comprises a monophyletic group including 6 classical species showing clonal evolution: *B. abortus*, *B. suis*, *B. melitensis*, *B. canis*, *B. ovis*, *and B. neotomae* (*1*,*2*). The zoonotic potential of *B. melitensis*, *B. abortus*, *B. suis*, and *B. canis* (in decreasing order of disease burden in human populations) has been clinically established on the basis of numerous human cases reported over the past century.

*B. neotomae* was originally isolated from a single rodent species (desert woodrat, *Neotoma lepida*), in an area with low population density of other wild animals and remote from domestic livestock (*3*). Recently, 2 publications described the isolation in Costa Rica of *B. neotomae* strains from 2 human patients with brucellosis (*4*,*5*). According to those reports, the 2 human isolates, bneohCR1 and bneohCR2, differed from each other by 164 single-nucleotide polymorphisms (SNPs); bneohCR1 differed from the *B. neotomae* genome used as reference in the analysis (GenBank accession no. GCA_000742255) by 174 and bneohCR2 by 160 SNPs. Those data indicated that *B. neotomae* has zoonotic potential and is present in a much wider geographic area than previously reported. 

Because that finding was unexpected and has substantial implications regarding our understanding of *Brucella*, we further investigated available information regarding the neglected species *B. neotomae*. We reviewed the literature for previous studies in which *B. neotomae* strains were isolated and searched public sequence repositories for *B. neotomae* whole-genome sequence (WGS) datasets. In addition, we identified and sequenced available *B. neotomae* strains maintained since the 1960s in 2 *Brucella* strain collections, the UK Animal and Plant Health Agency (APHA) Weybridge collection and the *Brucella* Culture Collection Nouzilly (BCCN) of the Institut National de l’Agriculture, de l’Alimentation et de l’Environnement (INRAE; National Research Institute for Agriculture, Food and the Environment) in France. We report a comprehensive comparative analysis of all genome sequences we identified from databanks and the human cases from Costa Rica, to further shed light on the genetic relationships between those isolates. 

## The Study

We recovered 17 *B. neotomae* WGS datasets from public repositories as assemblies or raw reads (last accessed May 31, 2023): ERR1894830, GCA_000158715, GCA_000712255, GCA_000742255, GCA_900446125, SRR004305, SRR004306, SRR032598, SRR857216, SRR4038991 (all 10 strains 5K33), ERR2993140 (MLVA31), GCA_900446115, SRR4038990 (5E1169), GCA_900446105 (6D152), ERR473742 (babohCR62), ERR1845156 (bneohCR2), and ERR1845155 (bneohCR1) (Appendix Table 2). We merged 3 records (SRR004305, SRR004306, SRR032598) corresponding to the same biosample. 

The *Brucella* strain collection maintained by APHA contained 5 and INRAE/BCCN, 7 *B. neotomae* strains (*6*,*7*). We recorded APHA and corresponding BCCN identifiers for each strain (Appendix Table 1). We produced and analyzed sequence data (Appendix). The 12 *B. neotomae* sequence datasets produced for this report were SRR22273182–8 (BCCN collection corresponding to primary names 6G152, 5E1169, 5E1266, 7E1260, 6H8988, and 5G239 and 1 unknown primary name) and SRR22414766–70 (APHA collection corresponding to primary names 7E164 and 5E1266 and 3 unknown primary names). We deposited sequences in the National Center for Biotechnology BioProject database as PRJNA901374 (BCCN) and PRJNA905663 (APHA) (Appendix Table 2). 

**Figure 1:**
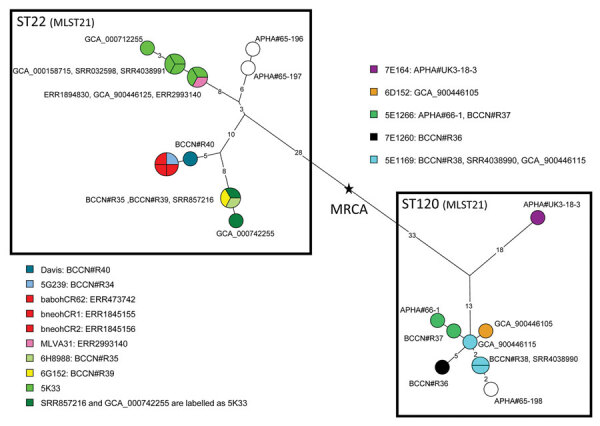
*Brucella neotomae* phylogeny. Maximum parsimony tree was derived from wgSNP data. We investigated 27 datasets and identified 149 SNPs; tree size is 151 substitutions (homoplasy 1.5%). Circles are colored according to primary strain identifier; red indicates the 3 datasets from Costa Rica. Circles are labeled with an accession number or collection strain identifier (*Brucella* Culture Collection Nouzilly [BCCN]) or Animal and Plant Health Agency [APHA] Weybridge collections). Branch lengths >1 substitution are indicated. Black star shows the position of the hypothetical MRCA. Box indicates the 2 MLST21 STs. MLST, multilocus sequence typing; MRCA, most recent common ancestor; SNP, single-nucleotide polymorphism; ST, sequence type; wgSNP, whole-genome single-nucleotide polymorphism.

We generated a maximum parsimony tree from the 149 SNPs identified among the 27 *B. neotomae* sequence datasets, including 15 public and 12 newly sequenced WGS datasets (Figure 1; Appendix Tables 1, 2). The whole-genome SNP (wgSNP) genotype of the most recent common ancestor (MRCA) of known *B. neotomae* lineages descends into 2 groups (Figure 1): the group containing type strain 5K33 corresponds to sequence type (ST) 22 in the *Brucella* multilocus sequence typing scheme MLST21, the other to ST120 (*7*). The limited available information about the sampling site of each strain from rodents in the Great Salt Lake Desert in Utah, USA, is consistent with congruence between *B. neotomae* phylogeny and the geography of the Great Salt Lake region, but further data are needed to robustly test this association (Appendix). 

**Figure 2:**
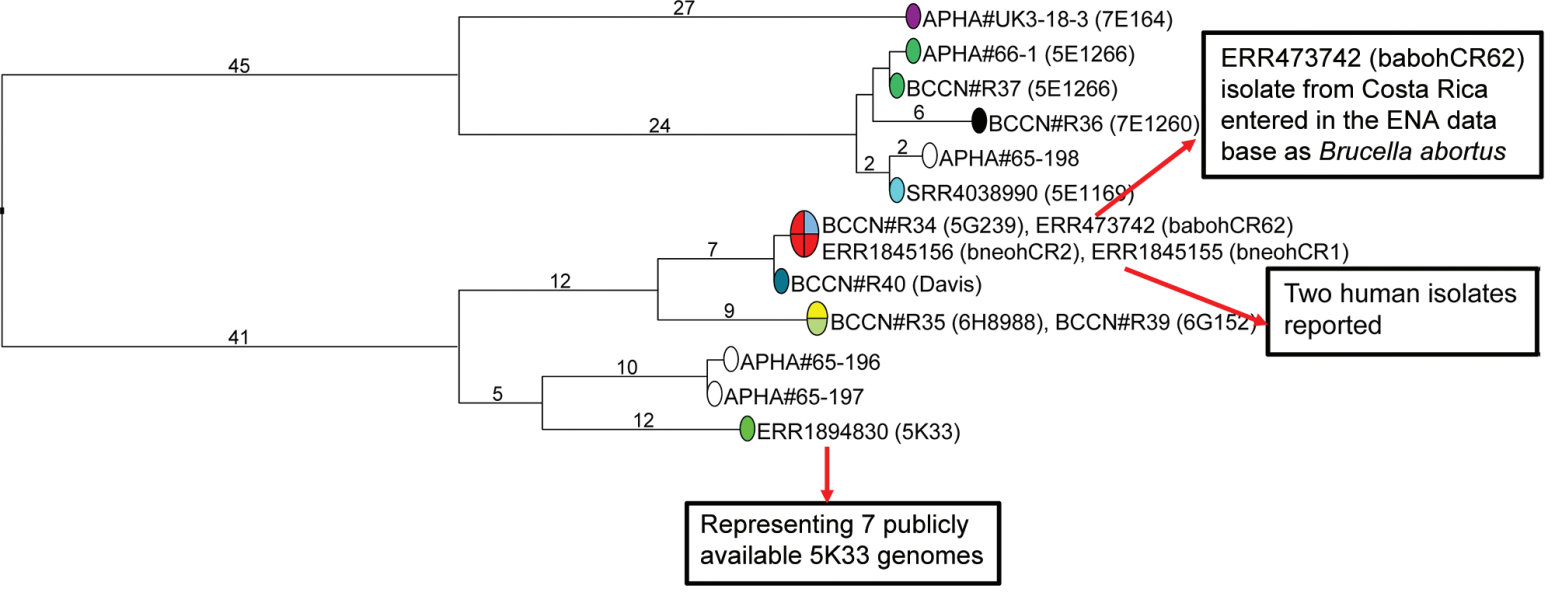
Rooted *Brucella neotomae* phylogeny of 16 selected datasets. Maximum parsimony tree was derived from wgSNP data; 205 SNPs in tree, tree size 207 substitutions (homoplasy 1%). Branch lengths >1 substitution are indicated. Circles are colored according to primary strain identity; red indicates the 3 datasets from Costa Rica. Circles are labeled with an accession number or collection strain identifier (*Brucella* Culture Collection Nouzilly [BCCN] or Animal and Plant Health Agency [APHA] Weybridge collections). Primary strain identifier is indicated in brackets when available. ENA, European Nucleotide Archive; SNP, single-nucleotide polymorphism; wgSNP, whole-genome SNP.

We show a different representation of the wgSNP phylogenetic analysis after removal of duplicates and of 1 dataset with relatively lower coverage (Figure 2; GenBank accession no. GCA_900446105 from strain 6D152). Because we removed the WGS datasets with partial coverage, the new tree contained 205 SNPs. The distances from MRCA to tips were similar: maximum 76 SNPs (to strain 7E1260) and minimum 56 SNPs (to strain APHA#65–197). The 3 whole-genome datasets from Costa Rica, including the human isolates bneohCR1 and bneohCR2 and the isolate babohCR62 entered as *B. abortus* in the European Nucleotide Archive database, remained identical in wgSNP genotype to strain 5G239 (BCCN#R34) in spite of the increased resolution. We still observed a coincident wgSNP genotype when we considered only these 4 strains, in sharp contrast with a report of human cases that indicated the corresponding genomes differed by 164 SNPs (*4*). 

## Conclusions

Our findings demonstrate that the strains isolated during 1955–1964 in the Great Salt Lake Desert in Utah display notable intraspecies genetic diversity despite being isolated from a geographically limited location, within a limited time frame, and from the same host species. In contrast, the datasets from wgSNP analysis of isolates from Costa Rica were identical despite having been isolated 4 years apart and in different areas of Costa Rica (*5*). Of note, datasets from analysis of isolates from Costa Rica were identical to data from 1 *B. neotomae* strain, 5G239, from the Great Salt Lake region. Finding an identical genotype in human cases from Costa Rica >3,000 km and >50 years apart in a different species from the Great Salt Lake discovery is remarkable in light of the diversity of strains noted in the geographically limited location in Utah and reported absence of rats of genus *Neotoma* in Costa Rica (*5*). Full understanding of the zoonotic potential of *B. neotomae* requires further exploration, including additional sampling of rodents and human cases in the US Southwest and Central America. 

Appendix Additional information about the genomic diversity and zoonotic potential of *Brucella neotomae*.
