# Supplementary material for: Genomic Diversity and Zoonotic Potential of Brucella neotomae
Source: Emerg Infect Dis. 2024 Jan;30(1):155–8. doi: 10.3201/eid3001.221783 (PMC10756370; doi:10.3201/eid3001.221783)
Supplement: Appendix — Additional information about the genomic diversity and zoonotic potential of Brucella neotomae. [file 22-1783-Techapp-s1.pdf]

Article DOI: https://doi.org/10.3201/eid3001.221783

EID cannot ensure accessibility for supplementary materials supplied by authors. Readers who have difficulty accessing supplementary content should contact the authors for assistance.

Genomic Diversity and Zoonotic Potential of *Brucella neotomae*

Appendix

Materials and Methods

The APHA and BCCN strains were sequenced independently using NextSeq and MiSeq sequencing machines (Illumina, https://www.illumina.com). Genome sequence assemblies were exchanged among the participating teams and phylogenetic analyses were run in duplicate.

Whole Genome Sequencing (WGS) and Data Analysis in France

Genomic DNA was extracted from the trypticase soy agar with 0.6% yeast extract (TSAYE)-cultured strains with the DNeasy blood and tissue kit (QIAGEN, https://www.qiagen.com). Library construction and WGS were performed by GenoScreen (https://www.genoscreen.fr) on an Illumina MiSeq platform, producing 250bp long paired-end reads. Sequencing reads were assembled using SPAdes version 3.13 (*1*). The assemblies had an average of 23 contigs (range 15–33), an average N50 of 458,544 (range 252,732–653,786) and an average total assembly length of 3.30 Mb.

Assemblies were imported into BioNumerics version 8.1 (https://www.applied-maths.com). The assemblies were used to produce artificial 50 bp reads, which were mapped for SNP calling on reference genome accession number GCA_000007125 (*B. melitensis* reference strain 16M) as previously described (*2*). Maximum parsimony analysis was used for phylogenetic reconstruction from whole genome SNP (wgSNP) data. Resulting trees were rooted using the reference *B. melitensis* 16M genome accession GCA_000007125 as outgroup.

Whole Genome Sequencing (WGS) and Data Analysis in UK

APHA strains were sequenced on the NextSeq platform, using the Illumina MiSeq v2 Reagent Kit, to produce 150 bp paired-end reads. Illumina data were assembled using Unicycler version v0.4.8, which implements SPAdes version 3.13.0 for *de novo* assembly of short-read data. The quality of genome assemblies was assessed using QUAST version 5.0.2. De novo assembly of five *B. neotomae* strains from APHA Weybridge produced genomes with an average of 33 contigs (range 31–36), an average N50 of 278,142 (range 222,135–294,583) and an average total assembly length of 3.30 Mb. Assembled genomes were used for subsequent phylogenetic analysis.

Publicly available *B. neotomae* WGS data were downloaded from EBI ENA (read archives) or NCBI (genome assemblies) (last updated 31/05/2023). Sequence data produced for this study were deposited in PRJNA905663 (APHA) or PRJNA901374 (BCCN) (Supplementary Table2).

Literature Review for *B. neotomae* Isolation

A search of PubMed for "*Brucella neotomae*" OR "*B. neotomae*" in Title/Abstract retrieved 78 publications (last updated 01/11/2023). These were published between 1958 and 2021. Prior to 2017, a single investigation was quoted leading to the isolation of *B. neotomae* strains, the original investigation in the Great Salt Lake Desert, Utah, USA first reported by Stoenner and Lackman in 1957 (*3*–*5*). The initial report indicated that five strains were isolated (*4*) and the last available report on this effort indicated that 16 strains were recovered by 1964 (*6*). Fifteen of these strains were isolated from the desert woodrat (*Neotoma lepida)* with the 16th isolated from a flea from the same host although unfortunately no strains names are provided in this summary publication.

The sixteen *B. neotomae* strains were recovered from eight sampling sites located within an area of approximately 130x160 km^2 (*6*). Three and five sites were located on the West and East sides of the desert, respectively and an equal number of strains was recovered from each side (*6*). We also identified ten strain names quoted in different publications and following the initial naming scheme (Appendix Table 1). We were not able to find the precise correspondence between strain identifiers and sites of isolation and only partial assignments could be inferred (*6*). The initial 1957 report (*3*) quoted five strains, 5K33, 5E1169, 5E1266, 6D152 and 5G239.

The list of locations and year of sampling (*6*) indicates that three of these five were collected in Gold Hill on the West side of the Great Salt Lake desert whereas two were collected in South Cedar Mountain on the East side. Additional names were subsequently quoted in the literature (Appendix Table 1). Strains 7E164 and 6H8488 first quoted in 1958 would have been sampled in Gold Hill (West side) and Little Davis Mountain (East side) without more precision. While the authors collected more than 6000 animals from 29 different species, all *B. neotomae* strains were recovered from the 258 desert woodrat representatives. Additionally, two other *Brucella* sp. were isolated from black-tailed jackrabbits (*Lepus californicus*). This suggests that for some unknown reason *B. neotomae* appeared to be surprisingly restricted to a single host within the sampling area (*7*).

In their review published in 2014 (*8*), Olsen and Palmer mention that "about 25 cultures of *B. neotomae* have been isolated" and quote the book published in 1988 by Alton et al. (*9*). This number refers to one sentence in the introduction « *B. neotomae* was isolated from the desert wood rat (*Neotoma lepida*), a rodent that inhabits western regions of the USA. Only about 25 cultures have been isolated, none of them from domesticated animals or man». The previous edition of this book was published in 1975 and mentioned that "27 cultures have been isolated" in an otherwise identical sentence (*10*). Among the authors, Lois Jones who had been working on *B. neotomae* strains at least since 1968 was likely well informed about the number of recovered strains (*11*). In 2017, two human cases with brucellosis due to *B. neotomae* were published (*12*,*13*).

Identification of *B. neotomae* Datasets in Public WGS Repositories

We recovered 4000 core *Brucella* WGS datasets including assemblies and sequence read archives. About 750 datasets were duplicates, due to WGS data deposited as assembly and sequence read archive, or due to the independent sequencing of classical strains (including type/reference strains and vaccines strains). Whole genome SNP analysis identified a cluster of 17 closely related WGS data sets, including all datasets from the *B. neotomae* 5K33 type strain. These 17 datasets correspond to 15 biosamples (Appendix Table 2). We merged the three datasets derived from the same biosample (SAMN00102852) after checking their coincidence in terms of wgSNP. Eight biosamples are registered as corresponding to the type strain 5K33, two as strain 5E1169, and one as strain 6D152. Three are reported as originating from Costa Rica and include biosample SAMEA2266954, described as *B. abortus* strain babohCR62*,* in addition to

the two human isolates bneohCR01 and bneohCR02 (*12*,*13*). The *B. abortus* sample data were made public in April 2014 whereas the human isolates data were made public in June 2017 at the time of the associated publications (*12*,*13*). It is notable that one of the human Costa Rican isolates, bneohCR01, was initially described as being (mis)identified as *B. abortus* on the basis of bacteriological and biochemical tests (*12*,*13*).

Results

Whole Genome SNP Analysis of *B. neotomae* WGS Data

Six out of eight datasets assigned to the 5K33 *B. neotomae* type strain clustered together as expected (Figure 1). The one to four SNPs observed among these 5K33 representatives presumably reflect variations resulting from laboratory cultivation of the type strain or sequencing errors. One outlier 5K33 assembly (GCA_000712255) results from the assembly of IonTorrent data (which are known to have a different error profile to Illumina sequence data) (*14*) and in the absence of the raw data we could not check the quality of the detected SNPs. The last two datasets labelled as 5K33 (GCA_000742255 and SRR857216) are more than 20 SNPs away from the 5K33 group and are identical to, or one SNP away from, two coincident strains, 6G152 and 6H8988 (Figure 1). The most parsimonious explanation for this finding is that these two datasets have been incorrectly labelled as being the *B. neotomae* 5K33 type strain. Strain "MLVA31" (ERR2993140 biosample SAMEA5176147, "imported case") is most likely a 5K33 representative since it is identical to two 5K33 datasets. The three datasets corresponding to 5E1169 were separated by two SNPs. SNP distances and tree topology in Figure 1 indicate that APHA#65-198 from the APHA Weybridge collection, and missing the original strain name, is most likely strain 5E1169.

Unfortunately, because of the lack of data regarding the sampling site of each strain, it was not possible for the present time to evaluate if genotypes circulate within the whole sampling area or if on the contrary the phylogenetic tree is congruent with the geographic origin within the Great Salt Lake desert sampling area recalled in the appendix. Two among the first five strains, 5K33 and 5G239, belong to MLST21 ST22 whereas three (5E1169, 5E1266, 6D152) belong to ST120. It is tempting to speculate that the ST22 strains originated from South Cedar Mountains on the East side, whereas the ST120 strains originated from Gold Hill on the

West side of the desert. The next two strains were isolated in 1956, Little Davis Mountains or 1957, Gold Hill and correspond to 7E164 and 6H8488 according to the list provided by (*6*) and to the first appearance of these strain names in the literature (Appendix Table1) (*15*). Strain 7E164 belongs to MLST21 ST120 where it defines a distinct lineage whereas 6H8988 belongs to ST22.

References

1. Bankevich A, Nurk S, Antipov D, Gurevich AA, Dvorkin M, Kulikov AS, et al. SPAdes: a new genome assembly algorithm and its applications to single-cell sequencing. J Comput Biol. 2012;19:455–77. PubMed https://doi.org/10.1089/cmb.2012.0021

2. Vergnaud G, Hauck Y, Christiany D, Daoud B, Pourcel C, Jacques I, et al. Genotypic Expansion Within the Population Structure of Classical *Brucella* Species Revealed by MLVA16 Typing of 1404 *Brucella* Isolates From Different Animal and Geographic Origins, 1974-2006. Front Microbiol. 2018;9:1545. PubMed https://doi.org/10.3389/fmicb.2018.01545

3. Stoenner HG, Lackman DB. A new species of *Brucella* isolated from the desert wood rat, *Neotoma lepida* Thomas. Am J Vet Res. 1957;18:947–51. PubMed

4. Stoenner HG, Lackman DB. A preliminary report on a Brucella isolated from the desert wood rat, *Neotoma lepida* Thomas. J Am Vet Med Assoc. 1957;130:411–2. PubMed

5. Stoenner HG, Holdenried R, Lackman D, Orsborn JS Jr. The occurrence of Coxiella burnetil, Brucella, and other pathogens among fauna of the Great Salt Lake Desert in Utah. Am J Trop Med Hyg. 1959;8:590–6. PubMed https://doi.org/10.4269/ajtmh.1959.8.590

6. Thorpe BD, Sidwell RW, Bushman JB, Smart KL, Moyes R. Brucellosis in Wildlife and Livestock of West Central Utah. J Am Vet Med Assoc. 1965;146:225–32. PubMed

7. Meyer ME. Evolution and taxonomy in the genus *Brucella*: Brucellosis of rodents. Theriogenology. 1976;6:263–72. https://doi.org/10.1016/0093-691X(76)90019-4

8. Olsen SC, Palmer MV. Advancement of knowledge of *Brucella* over the past 50 years. Vet Pathol. 2014;51:1076–89. PubMed https://doi.org/10.1177/0300985814540545

9. Alton GG, Jones LM, Angus RD, Verger JM. Techniques for the brucellosis laboratory. Paris, France: Institut National de la Recherche Agronomique (INRA); 1988.

10. Alton GG, Jones LM, Pietz DE. Laboratory techniques in brucellosis. 2nd ed. Geneva: World Health Organization; 1975.

11. Jones LM, Merz GS, Wilson JB. Phage typing reactions on *Brucella* species. Appl Microbiol. 1968;16:1179–90. PubMed https://doi.org/10.1128/am.16.8.1179-1190.1968

12. Suárez-Esquivel M, Ruiz-Villalobos N, Jiménez-Rojas C, Barquero-Calvo E, Chacón-Díaz C, Víquez-Ruiz E, et al. *Brucella neotomae* Infection in Humans, Costa Rica. Emerg Infect Dis. 2017;23:997–1000. PubMed https://doi.org/10.3201/eid2306.162018

13. Villalobos-Vindas JM, Amuy E, Barquero-Calvo E, Rojas N, Chacón-Díaz C, Chaves-Olarte E, et al. Brucellosis caused by the wood rat pathogen *Brucella neotomae*: two case reports. J Med Case Reports. 2017;11:352. PubMed https://doi.org/10.1186/s13256-017-1496-8

14. Salipante SJ, Kawashima T, Rosenthal C, Hoogestraat DR, Cummings LA, Sengupta DJ, et al. Performance comparison of Illumina and ion torrent next-generation sequencing platforms for 16S rRNA-based bacterial community profiling. Appl Environ Microbiol. 2014;80:7583–91. PubMed https://doi.org/10.1128/AEM.02206-14

15. Cameron HS, Meyer ME. Metabolic studies on *Brucella neotomae* (Stoenner and Lackman). J Bacteriol. 1958;76:546–8. PubMed https://doi.org/10.1128/jb.76.5.546-548.1958

16. Parnas J, Zalichta S, Sidor-Wójtowicz A. The taxonomic properties of *Brucellae* isolated from *Neotoma lepida* Thomas (*Brucella neotomae* Stoenner). Zentralbl Veterinärmed B. 1967;14:634–45. PubMed https://doi.org/10.1111/j.1439-0450.1967.tb00266.x

17. Meyer ME, Cameron HS. Comparative metabolism of species and types of organisms within the genus *Brucella.* J Bacteriol. 1959;78:130–6. PubMed https://doi.org/10.1128/jb.78.1.130-136.1959

18. Hall WH, Manion RE. In vitro susceptibility of *Brucella* to various antibiotics. Appl Microbiol. 1970;20:600–4. PubMed https://doi.org/10.1128/am.20.4.600-604.1970

19. Stoenner HG. Observations on the Behavior of *Brucella neotomae* in Laboratory Animals. Am J Vet Res. 1965;26:347–52.

20. Freeman BA, Baughn RE, McGhee JR. Some physical, chemical, and taxonomic features of the soluble antigens of the *Brucellae.* J Infect Dis. 1970;121:522–7. PubMed https://doi.org/10.1093/infdis/121.5.522

21. Beal GA, Lewis RE, McCULLOUGH NB, Claflin RM. Experimental infection of swine with *Brucella neotomae.* Am J Vet Res. 1959;20:872–5. PubMed

22. Stoenner HG. The behavior of *Brucella neotomae* and *Brucella suis* in reciprocal superinfection experiments in mice and guinea pigs. Am J Vet Res. 1963;24:376–80. PubMed

Appendix Table 1. List of *B. neotomae* strains identified in the literature and additional isolates identified in historical strain collections

Strain ID	Collection aliases	Quoting references	WGS data available	Comments
5K33	NCTC 10084<br>ATCC 23459	(*3*,*7*,*11*,*15*–*19*)	SRR032598[$]<br>SRR004305[$],<br>SRR004306[$]<br>SRR857216*<br>SRR4038991<br>GCA_000158715<br>GCA_000742255*<br>GCA_000712255<br>GCA_900446125<br>ERR1894830<br>ERR2993140[£]	[$] the three datasets correspond to the same biosample SAMN00102852, share the same wgSNP genotype and were subsequently merged under the name<br>* GCA_000742255 and SRR857216 do not cluster with the other 5K33 datasets<br>“SRR032598” in Figure 1<br>[£] MLVA31, biosample SAMEA5176147 incorrectly labelled “imported case, isolated in Germany” (Dr Enrico Georgi, personal communication)
5E1169	NCTC 10070,<br>BCCN#R38	(*3*,*7*,*11*,*15*,*16*,*19*)	SRR4038990<br>GCA_900446115<br>This report	BCCN strain transmitted by Dr L. Jones
5E1266	BCCN#R37<br>APHA#66-1	(*3*,*11*,*15*–*17*)	This report	Mistranscribed as SE1266 in (*16*). Received in APHA Weybridge collection in 1966.
6D152	NCTC 10071	(*3*,*15*,*16*,*19*)	GCA_900446105	
5G239	BCCN#R34	(*3*,*7*,*11*,*15*)	This report	Presumed mistranscribed as 56-239 in (*20*)
7E164	NCTC 10072,<br>APHA#UK3-18-3	(*7*,*11*,*15*,*18*,*19*,*21*,*22*)	This report	Presumed mistranscribed as 7E1164 in (*11*)
6H8488	BCCN#R35	(*7*,*11*,*15*)	This report	Presumed mistranscribed as 6H8988 in BCCN
6G152	BCCN#R39	(*7*,*11*)	This report	Presumed mistranscribed as 6G150 in (*11*) and 66-152 in (*20*). Transmitted by Dr L. Jones
7E1260	BCCN#R36	(*7*)	This report	
6D239		(*16*)		
Unknown	1963	(*7*)		A single strain was isolated in 1963,on the East side (*6*)
Unknown	239, 3n, 4n	(*16*)		
Unknown	BCCN#R40<br>(Davis)		This report	Transmitted by Dr L. Jones. Dr M. Meyer was working at University of California, Davis. Potential source? Possibly “1963” the only strain quoted by Meyer and missing in the BCCN and APHA Weybridge collections
Unknown	APHA#65-196	PubMLST	This report	Received in APHA Weybridge collection in 1965 from Dr Thorpe, Utah, USA. No additional identifier recorded.
Unknown	APHA#65-197	PubMLST	This report	Received in APHA Weybridge collection in 1965 from Dr Thorpe, Utah, USA. No additional identifier recorded
Unknown	APHA#65-198		This report	Received in APHA Weybridge collection in 1965 from Dr Thorpe, Utah, USA. No additional identifier recorded.

The original name of 8 among the 12 strains was recorded and corresponded to names identified in the literature except for 1 presumed transcription error.

Appendix Table 2 WGS datasets derived from *B. neotomae* strains

Key	BioProject	BioSample	Indicated strain	Country
ERR1894830	PRJEB19503	SAMEA103935299	5K33	USA
GCA_900446125	PRJEB6403	SAMEA104210778	NCTC10084 (5K33)	USA
GCA_900446115	PRJEB6403	SAMEA104318192	NCTC10070 (5E1169)	USA
GCA_900446105	PRJEB6403	SAMEA104318193	NCTC10071 (6D152)	USA
ERR473742	PRJEB4782	SAMEA2266954	babohCR62	Costa Rica
ERR1845156	PRJEB19503	SAMEA94360168	bneohCR02†	Costa Rica
ERR1845155	PRJEB19503	SAMEA94360918	bneohCR01†	Costa Rica
ERR2993140	PRJEB30030	SAMEA5176147	MLVA31	Germany
SRR004305	PRJNA33567	SAMN00102852	5K33	USA
SRR004306	PRJNA33567	SAMN00102852	5K33	USA
SRR032598	PRJNA33567	SAMN00102852	5K33	USA
SRR857216	PRJNA194124	SAMN01990992	5K33	USA
GCA_000712255	PRJNA230241	SAMN02427357	5K33	USA
GCA_000158715	PRJNA33567	SAMN02595289	5K33	USA
GCA_000742255	PRJNA243897	SAMN02768006	5K33	USA
SRR4038990	PRJNA251693	SAMN05417903	5E-1169	USA
SRR4038991	PRJNA251693	SAMN05417904	5K33	USA
SRR22273188*	PRJNA901374	SAMN31711931	5G-239	USA
SRR22273187*	PRJNA901374	SAMN31711932	6H-8988	USA
SRR22273186*	PRJNA901374	SAMN31711933	7E-1260	USA
SRR22273185*	PRJNA901374	SAMN31711934	5E-1266	USA
SRR22273184*	PRJNA901374	SAMN31711935	5E-1169	USA
SRR22273183*	PRJNA901374	SAMN31711936	6G-152	USA
SRR22273182*	PRJNA901374	SAMN31711937	Davis	USA
SRR22414766*	PRJNA905663	SAMN31880432	NCTC10072 (UK3/18-3) (7E164)	USA
SRR22414767*	PRJNA905663	SAMN31880431	66/1 (5E1266)	USA
SRR22414768*	PRJNA905663	SAMN31880429	65/197	USA
SRR22414769*	PRJNA905663	SAMN31880430	65/198	USA
SRR22414770*	PRJNA905663	SAMN31880428	65/196	USA

*This report
†Called bneohCR1 and bneohCR2 in reference (*12*).
